# The Effect of High-Intensity Power Training on Habitual, Intervention and Total Physical Activity Levels in Older Adults with Type 2 Diabetes: Secondary Outcomes of the GREAT2DO Randomized Controlled Trial

**DOI:** 10.3390/geriatrics6010015

**Published:** 2021-02-08

**Authors:** Marjan Mosalman Haghighi, Yorgi Mavros, Shelley Kay, Kylie A. Simpson, Michael K. Baker, Yi Wang, Ren Ru Zhao, Jacinda Meiklejohn, Mike Climstein, Anthony J. O’Sullivan, Nathan De Vos, Bernhard T. Baune, Steven N. Blair, David Simar, Nalin Singh, Jeffrey Schlicht, Maria A. Fiatarone Singh

**Affiliations:** 1 Faculty of Medicine and Health, University of Sydney, Camperdown 2050, Australia; yorgi.mavros@sydney.edu.au (Y.M.); k_y_l_i_e@hotmail.com (K.A.S.); rzha6475@uni.sydney.edu.au (R.R.Z.); Jacindawm@gmail.com (J.M.); Michael.Climstein@scu.edu.au (M.C.); nsingh@strong-medicine.com.au (N.S.); maria.fiataronesingh@sydney.edu.au (M.A.F.S.); 2 Centre for Medical Psychology and Evidence Based Decision Making, Faculty of Medicine, University of Sydney, Camperdown 2050, Australia; shelley.kay@sydney.edu.au; 3 School of Behavioural and Health Sciences, Australian Catholic University, Strathfield 2135, Australia; Michael.Baker@acu.edu.au; 4 Lipid Metabolism & Cardiometabolic Disease Laboratory, Baker Heart and Diabetes Institute, Melbourne 3004, Australia; Yi.Wang@baker.edu.au; 5 Clinical Rehabilitation Research Centre, University of Longyan, Longyan 364012, China; 6 School of Health and Human Sciences, Southern Cross University, Gold Coast 4225, Australia; 7 Department of Endocrinology, Faculty of Medicine, University of New South Wales, Sydney 2052, Australia; a.osullivan@unsw.edu.au; 8 The Centre for STRONG Medicine, Balmain Hospital, Balmain 2041, Australia; Nathan.DeVos@health.nsw.gov.au; 9 Department of Psychiatry, University of Muenster, 48149 Muenster, Germany; Bernhard.Baune@ukmuenster.de; 10 Department of Psychiatry, Melbourne Medical School, The University of Melbourne, Melbourne 3010, Australia; 11 The Florey Institute of Neuroscience and Mental Health, The University of Melbourne, Parkville 3010, Australia; 12 Exercise Science Arnold School of Public Health, University of South Carolina, Columbia, SC 29208, USA; SBLAIR@mailbox.sc.edu; 13 School of Medical Sciences, Faculty of Medicine, University of New South Wales, Sydney 2052, Australia; d.simar@unsw.edu.au; 14 Department of Health Promotion and Exercise Sciences, Western Connecticut State University, Danbury, CT 06810, USA; schlichtj@wcsu.edu; 15 Sydney Medical School, University of Sydney, Camperdown 2050, Australia; 16 Jean Mayer USDA Human Nutrition Research Center on Aging, Tufts University, Boston, MA 02129, USA

**Keywords:** physical activity change behavior, metabolic profile, PASE score, HbA1c, HOMA-IR

## Abstract

Background: We examined the effect of power training on habitual, intervention and total physical activity (PA) levels in older adults with type 2 diabetes and their relationship to metabolic control. Materials and Methods: 103 adults with type 2 diabetes were randomized to receive supervised power training or sham exercise three times/week for 12 months. Habitual, intervention, and total PA, as well as insulin resistance (HOMA2-IR) and glycosylated hemoglobin (HbA1c), were measured. Results: Participants were aged 67.9 ± 5.5 yrs, with well-controlled diabetes (HbA1c = 7.1%) and higher than average habitual PA levels compared to healthy peers. Habitual PA did not change significantly over 12 months (*p* = 0.74), and there was no effect of group assignment on change over time in habitual PA over 0–6 (*p* = 0.16) or 0–6–12 months (*p* = 0.51). By contrast, intervention PA, leg press tonnage and total PA increased over both 6- and 12-month timepoints (*p* = 0.0001), and these changes were significantly greater in the power training compared to the sham exercise group across timepoints (*p* = 0.0001). However, there were no associations between changes in any PA measures over time and changes in metabolic profile. Conclusion: Structured high-intensity power training may be an effective strategy to enhance overall PA in this high-risk cohort.

## 1. Introduction

Physical activity (PA) is a powerful non-pharmacological treatment for the management of impaired glucose tolerance and diabetes [1,2], yet individuals with type 2 diabetes are less likely to engage in PA than age-matched peers without diabetes [3,4], and this disparity has been shown to increase with age. Participation in supervised exercise may address physical and psychological barriers to PA in older adults with type 2 diabetes, promoting increased habitual PA.

However, previous studies of aerobic exercise [5,6,7,8] have reported that participants displace habitual PA (activity not prescribed by the study protocol) with study PA, resulting in no change in total PA. There are limited empirical data testing the effect of progressive resistance training (PRT) on habitual PA.

Only four randomized control trials (RCTs) have reported the effect of supervised PRT on habitual PA in older adults with diabetes. Dunstan et al., 2002 [9] and Church et al., 2010 [10] investigated self-reported and objective changes in habitual PA, respectively, after long-term (>6 months) PRT + hypocaloric diet and aerobic, PRT, or aerobic + PRT training, respectively, reporting no significant differences between intervention and control groups. By contrast, Castaneda et al., 2002 [11] and Balducci et al., 2010 [12] documented increases in self-reported habitual PA after 16 weeks of PRT and 12 months of aerobic + PRT training, respectively. None of these studies used physically active control groups for comparison, although Dunstan’s control group did low-intensity stretching exercises. Thus, additional robust investigations are needed to understand whether exercise interventions influence habitual PA levels in this and other cohorts.

Older adults with type 2 diabetes have a higher prevalence of obesity, muscle dysfunction, mobility impairment, poor balance, cardiovascular disease, peripheral neuropathy and depression, all of which may impede engagement in PA [13,14,15]. These common comorbidities provide a rationale for prescribing supervised exercise to individuals with type 2 diabetes. However, not all exercise has the potential to robustly address these barriers. High-intensity power training, a high velocity, high-intensity form of PRT, improves muscle power [16], functional performance [17] and balance [18] more effectively than slow velocity PRT, while providing similar increases in muscle strength and endurance [19,20]. In addition, power training is a more potent stimulus for fast-twitch muscle fibres which are preferentially atrophied/lost during age-related sarcopenia [21,22] and myopathy associated with type 2 diabetes [23]. Thus, while power training may potentially overcome many barriers to increased habitual PA in this cohort, this mode of exercise training has never been investigated with regards to this outcome. Therefore, we hypothesized that power training would increase habitual, intervention and total (intervention plus habitual) PA levels compared to controls over 12 months in older adults with type 2 diabetes, and that these increases would be associated with improvements in metabolic profile.

## 2. Materials and Methods

The Graded Resistance Exercise And Type 2 Diabetes in Older Adults (GREAT2DO) Study was a 12-month randomized, double-blind, sham exercise-controlled trial (RCT)- Australian New Zealand Clinical Trial Registry. no. 12606000436572- with a 5-year follow-up period which tested the efficacy of power training added to the usual medical care of older adults with type 2 diabetes and metabolic syndrome [24]. These analyses pertain to the initial 12-month intervention. Detailed methods have been described previously [24,25] and are presented briefly below.

### 2.1. Study Subjects

Participants were recruited from August 2006 to November 2009. Participants were included if they were ≥ 60 years, previously diagnosed with type 2 diabetes, had a body mass index (BMI) > 25 kg/m^2^, were sedentary (no progressive resistance training; structured exercise ≤ 1/week; less than 150 min/week of moderate–vigorous-intensity walking or other aerobic exercises) and had metabolic syndrome [26]. Exclusion criteria were significant cognitive impairment, non-ambulatory status, lower-extremity amputation other than toes, current alcohol or substance abuse, unstable cardiovascular disease, proliferative diabetic retinopathy, inability to adhere to study requirements over the course of 1 year due to travel plans or other commitments, or rapidly progressive terminal illness. Written informed consent was obtained, and the protocol was approved by the Sydney South West Area Health Service and the University of Sydney Human Research Ethics Committees (Australian New Zealand Clinical Trial Registry. no. 12606000436572).

### 2.2. Training Protocol

Supervised high-intensity power training was carried out 3 days/week for 12 months (approximately 45 min/session) using pneumatic resistance equipment (Keiser Sports Health Ltd., Fresno, CA, USA). Concentric contractions were completed as quickly as possible (<1 s); eccentric contractions were completed over 4 s. Exercises included bilateral chest press, seated row, knee extension and leg press, and unilateral standing hip abduction, hip flexion and hip extension. Participants performed 3 sets of 8 repetitions for each exercise, except for the hip exercises (2 sets of 8 reps for each leg). Intensity was set at 80% of the most recently determined 1-repetition maximum (1RM). Where 1RM testing was not feasible, loads were increased by targeting a Borg Scale rating of perceived exertion between 15–18, which has been shown to approximate 80% 1RM [27]. The 1RMs were reassessed every 4 weeks.

Supervised SHAM exercise training utilized the same equipment (3 times/week, 3 sets of 8 reps, for approximately 30 min) and trainers. Resistance was set to the lowest limit of each machine and not progressed, and participants were instructed to perform concentric and eccentric contractions slowly (over approximately 3 s). Both interventions were presented as potentially beneficial and took place at separate times of day so participants remained blinded to investigators’ hypotheses.

For both groups, rest periods up to 1–2 min between sets and exercises were inserted as needed to ensure proper form and minimize fatigue.

### 2.3. Habitual Physical Activity Level

Habitual PA was assessed using the Physical Activity Scale for the Elderly (PASE). The PASE is a brief instrument designed to assess PA in older adults over a 1-week time frame [28]. The questionnaire is comprised of self-reported occupational, household and leisure activities items and was administered by a blinded assessor. All study participants were asked to avoid any new structured exercise during the initial 12 months of the intervention, but any non-structured activity was allowed as desired. Habitual, intervention and total PA levels were extracted from the PASE at baseline, 6 and 12 months.

#### Physical Activity Level Definitions

The measures below were extracted from the PASE: (see Table 1)

1. Habitual PA = any activity performed outside intervention sessions.
2. Intervention PA = Power training sessions defined as muscle strength/endurance exercise and SHAM defined as light intensity exercise [29,30].
3. Total PA = [average number of intervention sessions attended over the previous 6-months (% attendance) × 3 d/week × duration of session (h/day) × weighting factor (30 for power sessions and 21 for sham sessions)] + habitual PA.
  - % attendance at 6 months = average attendance over the first 6 months of intervention.
  - % attendance at 12 months = average attendance over months 6–12.

Index of tonnage = summing of force (N) on the leg press machine across every session/force at the 4th training session.

Total leg press relative tonnage (over 6 or 12 months) = Index of tonnage × number of repetitions × number of sets.

Example calculations for study exercise:

For the power training group, study exercise score = % attendance in the previous 6 months × 3 times/week × 45 min/session × PASE muscle strength/endurance exercise weighting of 30.

For the sham group, study exercise score = % attendance in the previous 6 months × 3 times/week × 30 min/session × PASE light intensity exercise weighting of 21.

We also used a more precise measurement for intervention PA which was total tonnage on the leg press during training. This was calculated by first summing for each participant the amount of force (N) set on the leg press machine across every session they attended, and then dividing that sum by the force used at their 4th training session (when the participants had been progressed to their intended 80% 1RM load), to create an index of tonnage normalized to each participant’s baseline strength. This ratio was then multiplied by the number of repetitions × number of sets of training to represent total leg press relative tonnage over the 6 or 12 months. We used leg press tonnage as it represented the machine that was available for most participants and involved the major muscle groups of the lower body. This process was carried out for both the power training and sham group in the same manner. By design, the forces were not increased over time in the sham group, so variations in tonnage would have been primarily related to attendance rate. By contrast, the variable increase in tonnage in the power training group depended on factors including attendance rate, ability to adhere to the intended 80% intensity, and capacity for anabolic adaptation to the stimulus. In other studies, training tonnage has been used as an index of the anabolic benefits of exercise for body composition outcomes such as bone density [31], but has not previously been investigated as a marker for metabolic outcomes to our knowledge.

### 2.4. Measures of Insulin Resistance and Glucose Homeostasis

Serum glucose, C-peptide, and glycosylated hemoglobin (HbA1c) were measured by a commercial pathology laboratory using standard assays (Douglass Hanley Moir, Sydney, Australia). Homeostatic model assessment insulin resistance (HOMA2-IR) was calculated with plasma glucose and C-peptide using the validated calculator (accessed at http://habitual.dtu.ox.ac.uk) [32]. The 12-h fasted HOMA2-IR was measured in the morning, 96 h post-exercise, after a brief period of supine rest in the clinic. Insulin/hypoglycemic medications were halted 12 h prior to phlebotomy. Sixteen insulin-treated participants were excluded from analyses, as HOMA2-IR has not been validated in this cohort [33].

### 2.5. Anthropometrics

Morning fasting stretched stature (wall-mounted Holtain stadiometer; Holtain Limited, Crymych SA41 3UF, UK) and naked body mass were measured to the nearest 0.1 cm and 0.01 kg, respectively, as the average of three measurements. Body mass index (BMI) was calculated by dividing naked body mass by height^2^ (kg/m^2^). Waist circumference was measured according to the International Diabetes Federation (IDF) protocol [26] with a Lufkin steel tape measure (W606 PM, Apex Tool Group Pty Limited, Albury, NSW, Australia).

## 3. Statistical Analyses

All data were assessed for normality visually and statistically. Normally and non-normally distributed data are presented as mean ± standard deviation (SD) and median (range), respectively. Non-normally distributed data were log-transformed for use with parametric statistics where possible. Linear mixed models [34] with repeated measures were used to investigate the effect of Time and the Group × Time interaction for all three PA outcomes (as defined above) over 0 and 6 and over 0, 6 and 12 months. If there was a significant Time or Group × Time interaction in any mixed model, Bonferroni post-hoc t-tests were used to identify significant differences. Potential confounders entered into the mixed models as covariates were baseline age and HbA1c, which were slightly different between power training and SHAM, and waist circumference which was significantly correlated with habitual PA at baseline.

Next, regression models were constructed to investigate hypothesized relationships between changes in PA and metabolism. Change of metformin dosage, waist circumference and baseline age were entered into all multiple regression models along with all PA measures and leg press tonnage as potential independent predictors of HbA1c and HOMA2-IR. Separate models were constructed for each type of PA and tonnage and each metabolic outcome. Age and waist circumference were entered because previous studies [35,36,37] have shown that age and central obesity are related to metabolic profile. SPSS (Version 24, SPSS Inc., Chicago, IL, USA) was used for all data analysis. A *p*-value < 0.05 was accepted as the threshold for statistical significance as all hypotheses were specified a priori.

## 4. Results

**Figure 1:**
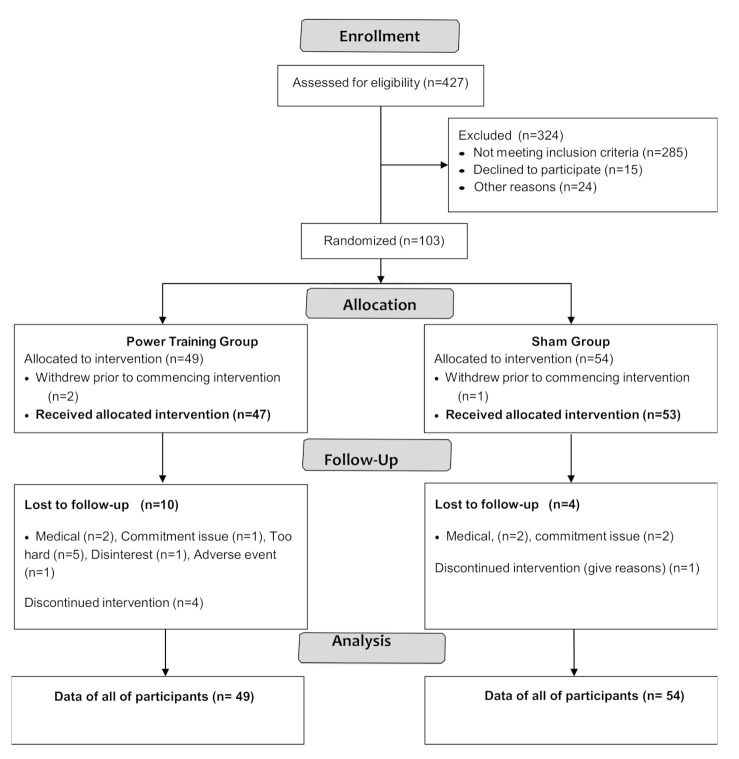
CONSORT 2010 Flow Diagram.

Participant flow through the study is shown in the CONSORT flowchart (Figure 1).

Participant characteristics are shown in Table 2. PASE scores (stratified by age group and sex) were compared to normative data from healthy peers [29] by using a one-sample *t*-test. The results showed there were no significant differences between PASE scores of our participants and healthy older adults except that women aged 70–75 in GREAT2DO were significantly more active than healthy peers [29] (data are not shown).

Among 103 participants enrolled, three withdrew prior to intervention and 16 dropped out. Adherence to exercise training was 76 ± 15% and 79 ± 15% in the power training and SHAM groups, respectively, with no significant difference between groups (*p* = 0.35).

### 4.1. Changes in PA Level

#### 4.1.1. Habitual PA Score

**Figure 2:**
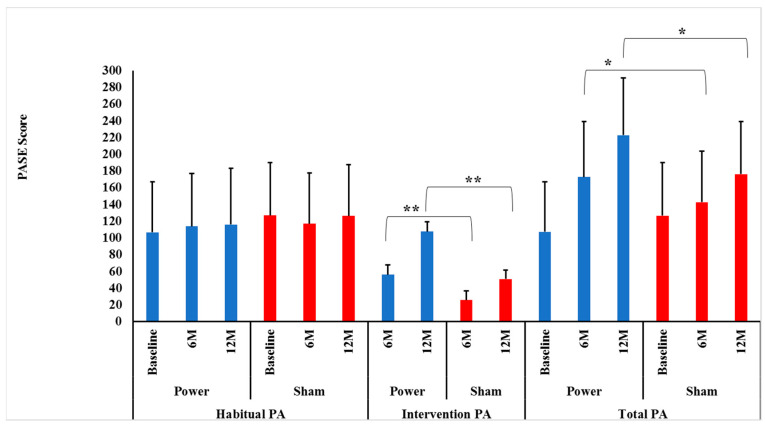
Intervention, habitual and total physical activity level between groups by time. Data presented as mean ± SD. M = months, Power = power training group (BLUE bars), SHAM = control group (RED bars). There were no significant Group×Time interactions for habitual physical activity (PA) between power and SHAM groups over 0–6 or over 0–6–12 months (*p* = 0.16 and *p* = 0.51, respectively). ** There were significant Group × Time interactions for intervention PA between power and SHAM groups over both 0–6 and over 0–6–12 months (*p* = 0.0001 and *p* = 0.0001, respectively). Bonferroni post-hoc *t*-tests showed power training had a higher intervention PA at both 6 (*p* = 0.0001) and 12 months (*p* = 0.0001) compared to SHAM. * There were significant Group × Time interactions for total PA between power and SHAM groups over 0–6 and over 0–6–12 months (*p* = 0.0001 and *p* = 0.0001, respectively). Bonferroni post-hoc *t*-tests showed power training had a higher intervention PA at both months (*p* = 0.02) and 12 months (*p* = 0.0001) compared to SHAM.

Contrary to our hypotheses, habitual PA did not change differentially between the power training and SHAM groups over 0–6 (*p* = 0.16) or 0–6–12 months (*p* = 0.51), indicating that group assignment did not influence habitual PA over time. In addition, there were no significant Time effects over 0–6 (*p* = 0.81) or 0–6–12 months (*p* = 0.74), indicating that habitual PA was maintained over time (Figure 2).

#### 4.1.2. Intervention PA/Tonnage Score

As hypothesized, there were significant Group × Time interactions between power and SHAM groups over both 0–6 and 0–6–12 months (*p* = 0.0001 and *p* = 0.0001, respectively) for intervention PA. Bonferroni post-hoc *t*-tests identified that the power training group had higher intervention PA at both 6 (*p* = 0.0001) and 12 months (*p* = 0.0001) compared to SHAM (Figure 2). This sustained improvement in intervention PA power training versus SHAM indicates there was no attenuation of adherence or progression in the high-intensity power training cohort, contrary to many long-term exercise studies’ findings [38,39].

Again, as hypothesized, there were significant Group × Time interactions between power training and SHAM groups for leg press tonnage over both 0–6 and 0–6–12 months (*p* = 0.0001 and *p* = 0.0001, respectively). Bonferroni post-hoc *t*-tests identified that the power training group had higher leg press tonnage at both 6 (*p* = 0.0001) and 12 months (*p* = 0.0001) compared to SHAM, indicating adherence to progressive intensity protocols were maintained over the year (Figure 2).

#### 4.1.3. Total PA Score

As hypothesized, there were significant Group × Time interactions between power training and SHAM over both 0–6 and 0–6–12 months (*p* = 0.0001 and *p* = 0.0001, respectively). Bonferroni post-hoc *t*-tests revealed that the power training group had higher total PA at both 6 (*p* = 0.02) and 12 months (*p* = 0.001) compared to SHAM (Figure 2). Thus, over the entire 12 months, there was no indication that habitual PA was replaced by intervention PA, as maintained habitual PA levels in conjunction with increased intervention PA led to the intended higher Total PA in the power training group.

Relationships between Changes in Habitual, Intervention and Total PA Levels, Leg Press Tonnage, and Metabolic Profile.

Contrary to our hypotheses, there were no significant associations between changes in any PA measure and changes in either HbA1c or HOMA2-IR over 0–6 or 0–12 months for either the power training or SHAM groups (r = 0.04–0.2; *p* = 0.3–0.82).

## 5. Discussion

To our knowledge, this is the first power training intervention in older adults with type 2 diabetes, as well as the first to investigate the effects of this modality on habitual, intervention and total PA levels. Power-trained participants significantly increased intervention PA, total tonnage, and total PA levels, but were not more habitually physically active compared to SHAM participants. Importantly, power trainers did not replace habitual PA with intervention PA, such that total PA increased significantly at both 6 and 12 months. This is an important long-term benefit, given the substantial amount of data in this cohort documenting PA declines over time. Unexpectedly, no PA outcomes were related to changes in metabolic profile.

### 5.1. Habitual PA Level

#### 5.1.1. Why Habitual PA Did Not Increase over Time

Contrary to our hypothesis, power training did not increase habitual PA engagement relative to baseline or SHAM. However, in contrast to typical age-related decreases in PA over time, or PA decreases reported during aerobic interventions [6], both groups maintained their habitual PA levels. There are several possible explanations for these findings. First, power-trained participants engaged in 125 min of vigorous exercise per week, which met national PA recommendations [40]. Therefore, they may not have perceived the need for additional PA. This is similar to what was seen by Ehlers et al. [41] who utilized moderate-to-vigorous intensity aerobic exercise. Second, participants were more active at baseline than expected [29], and had few hypothesized barriers to habitual PA expected in older adults, such as sarcopenia, mobility impairment, peripheral neuropathy, depression, or cognitive impairment. This may be attributed to selection bias common in clinical exercise trials where healthier people tend to volunteer, as well as the study’s exclusion criteria (significant cognitive impairment, inability to travel to the venue, non-ambulatory status or unstable disease). By contrast, in a study where resistance training did improve habitual PA by 34% [42], participants were nursing home residents with many barriers to habitual PA: mean age 87 years (38% >90 years); 83% required a cane, walker, or wheelchair; 66% had fallen during the previous year; 40–50% had arthritis, pulmonary disease, osteoporotic fracture, and cognitive impairment/depression, and they had a baseline PA about 25% of PA levels of sedentary young adults. Third, and critically, all participants were told not to add any new structured exercise while they were in the intervention phase. Therefore, the only changes in habitual PA that could have occurred and been captured on the PASE would have been in the domains of incidental walking outside of the house, housework, repairs, yard-work/gardening, caregiving duties, volunteer or paid work involving PA.

#### 5.1.2. Why Habitual PA Did not Decrease over Time

Age, disease, and obesity in our cohort would be predicted to decrease habitual PA over time, particularly in SHAM participants who were not doing exercise that would increase physiological reserve. In addition, time constraints imposed by the study could have led individuals to stop or reduce doing certain activities in daily life, thus reducing habitual PA. However, we did not observe decreases in habitual PA in either group. This may be because the supervised gym-based exercises were novel activities that could not simply substitute for something similar in real life. Similarly, in the only study to directly compare aerobic, resistance and combined training in older adults with diabetes [10], habitual PA was preserved in all groups (Table 3). By contrast, substitution has occurred in aerobic exercise trials [5,6,7,8]. For example, in a non-RCT (n = 11 older adults), participants performing vigorous endurance training had no significant change in total energy expenditure because they decreased their habitual PA by 62% [6]. Further direct comparisons of more impaired cohorts using varying exercise modalities and intensities of supervision are required to confirm and extend our findings.

#### 5.1.3. Intervention PA and Feasibility of High-Intensity Power Training

Adherence rates were similar and high in both groups for 12 months. This contradicts the suggestion made by Perri et al., [43] that older adults may have low adherence to high-intensity exercise prescription. This robust, anabolic and novel intervention was highly feasible in this older cohort with multiple comorbidities. Many investigators, including Eves et al., 2006 [44], Dunstan et al., 2002 [9], Sigal et al., 2007 [45], Castaneda et al., 2002 [11] and Balducci et al., 2010 [12] among others, have reported that PRT is safe and well-tolerated with similar clinical benefits to aerobic exercise in people with type 2 diabetes. PRT is a desirable alternative for participants who cannot engage sufficiently in aerobic exercise due to co-morbidities such as recurrent falls, osteoarthritis or claudication.

Our study is the first to report that the intensity of training reached 80% of the 1RM by the 4th week of the trial (i.e., after only nine sessions—three sessions/week × three weeks). It should be noted that perceived exertion was obtained every training session on every machine, both subjectively (from the participant) and objectively (from the judgement of the expert trainer as to the level of effort being expended by the participant) and was used to set the load for that day. This rigorous process optimized the continuous progression of loading and maintenance of the intended intensity prescription. The significant increase in leg press tonnage reported at 12 months compared to 6 months is an objective measurement of adherence to the progressive nature of the intervention. Castaneda et al. 2002 [11], reported participants reached 80% of baseline 1RM after 8 weeks while Dunstan et al., 2002 [9] reported intensity of training was 50–60% of the baseline 1RM during weeks 1–2 with a goal to achieve between 75 and 85% over time, but they did not report if or when participants reached this goal. Thus, several prior RCTs, and this study, in particular, have now shown that high-intensity PRT is feasible in older adults with type 2 diabetes.

### 5.2. Total PA Level

We observed an increase in total PA in power training compared to SHAM, which was due solely to the increase in intervention PA, as habitual PA did not increase. These results are identical to those reported by Dunstan et al., 2002 [9] and Church et al., 2010 [10]. In addition, Castaneda [11] and Balducci [12] reported an increase in habitual PA in their cohorts after resistance and combined training, respectively. Taken together, these studies suggest that PRT is a potentially important way to increase total PA while maintaining or even increasing habitual PA in overweight/obese older adults with type 2 diabetes.

#### Associations among PA Levels and Metabolic Profile

There were no significant group differences between changes in any PA measure and decreases in HbA1c or HOMA2-IR. However, participants in our study had well-controlled HbA1c levels with a mean of 7.1% (54 mmol/mol) at baseline, which provided little room for improvement due to a “floor effect”. We may have found more significant results among participants with poorer glycemic control, such as those in Castaneda’s study [11].

We could not control usual care provided by physicians outside the study protocol, who may have responded to elevations or reductions of glucose with alterations to medications and/or the introduction of insulin in both groups over the course of 12 months, thus blunting our ability to discern the effects of exercise alone.

We only controlled for changes in metformin dosage in regression models. It is possible that control for other hypoglycemic medications and insulin dosages was needed to eliminate the effects of changes in medical therapy. The large number of different drugs and dosages used by our subjects precluded this approach.

Previous literature suggests that the relationship between glucose homeostasis and PA levels may depend on the definition of the PA intervention used. For example, in our meta-analysis of long term (at least 6 months in duration) PA interventions in this cohort [43], which included 14 RCTs of either PA promotion or supervised/unsupervised exercise interventions, we reported no significant association between habitual PA levels and HbA1c (r= −0.46, *p* = 0.64). By contrast, Umpierre et al., in a systematic review of RCTs in this cohort [2] reported that supervised, structured aerobic, PRT, or combined exercise for at least 12 weeks significantly reduced HbA1c to a similar extent as the addition of a new hypoglycemic medication (−0.67% on average), whereas simple behavioural advice to exercise without dietary advice was not effective.

The reason for the lack of relationship of PA changes to IR could be that HOMA2-IR represents hepatic insulin resistance, rather than skeletal muscle insulin resistance, and it is likely that this measure is insufficiently sensitive to beneficial adaptations occurring in muscle metabolism after exposure to anabolic exercise such as power training. In addition, improvements in insulin sensitivity after acute exercise are known to wane within 48–72 h, so it is possible that our measurements, taken at 96 h after the last exercise bout (to capture chronic training adaptations), may not have represented the full effects of intermittent bouts of exercise or habitual PA in the follow-up period.

## 6. Limitations

Limitations include selection bias of cohort, which may limit generalization to frail or more impaired older adults with long-standing type 2 diabetes and many co-morbidities. Subjective measurement of PA with the PASE questionnaire involves recall and other biases, and the PASE has low sensitivity for capturing low levels of PA [46], which means we may not have captured all incidental PA. Objective PA assessment may have improved the ability to capture small changes in low-intensity incidental PA.

## 7. Conclusions

Habitual PA levels were maintained in older adults with type 2 diabetes who were instructed not to take up other forms of structured exercise during 12 months of supervised high-intensity power training or low-intensity SHAM exercise. Because power training was associated with significantly greater intervention PA and leg press tonnage over 12 months, our intended increases in total PA relative to SHAM exercise controls was achieved. There is a need for additional research investigating long-term PA changes measured objectively during and after exposure to structured anabolic vs. aerobic exercise and their associations with health status outcomes in people with type 2 diabetes. Targeting vulnerable individuals with greater deficits in functional performance, mobility and muscle function than those studied to date may enhance detection of potential clinical benefits of robust exercise in this cohort.

## Figures and Tables

**Table 1:** Physical Activity Scale for the Elderly (PASE).

PASE Activity	PASE Weight	Activity Frequency	PASE Score = Weight × Frequency
Muscle strength/endurance	30		
Strenuous sports	23		
Moderate sports	23		
Light sports	21		
Job involving standing/walking	21		
Walk outside	20		
Lawn work or yard care	36		
Caring for another person	35		
Home repairs	30		
Heavy housework	25		
Light housework	25		
Outdoor-gardening	20		
**PASE Total**			

**Table 2:** Baseline characteristics.

Demographics and Health Status	Group	Mean ± SD or N, (%)
Age (years)	Power	67.0 ± 5.0
	SHAM	69.0 ± 6.0
	Total	67.9 ± 5.5
Men, Women (n)	Power	25, 24
	SHAM	27, 27
Duration of diabetes (years)	Power	7 ± 5
	SHAM	9 ± 7
	Total	8.0 ± 6.0
Average stretch stature (m)	Power	1.7 ± 0.1
	SHAM	1.7 ± 0.1
	Total	1.7 ± 0.1
Weight (kg)	Power	90.0 ± 15.3
	SHAM	88.7 ± 18.8
	Total	89.1 ± 17.1
BMI (kg/m^2^)	Power	31.5 ± 4.6
	SHAM	31.6 ± 6.0
	Total	31.6 ± 5.4
Waist circumference (cm)	Power	110.1 ± 11.9
	SHAM	109.0 ± 12.4
	Total	109.5 ± 12.1
Total no. chronic diseases (n)	Power	5 ± 2
	SHAM	5 ± 2
	Total	5 ± 2
Osteoarthritis, n (%)	Power	35 (71.4)
	SHAM	33 (61.1)
	Total	68.0 (66.0)
Diabetic treatment, n (%)		
Diet only	Power	8 (16)
	SHAM	10 (19)
	Total	18 (17)
Oral hypoglycemics only	Power	34 (69)
	SHAM	35(65)
	Total	69 (67)
Oral hypoglycemics + insulin	Power	4 (8)
	SHAM	7 (13)
	Total	11 (11)
Insulin only	Power	3 (6)
	SHAM	2 (4)
	Total	5 (5)
Metabolic profile		
HbA1c (%)	Power	6.9 ± 0.9
	SHAM	7.3 ± 1.3
	Total	7.1 ± 1.1
Fasting serum glucose (mmol/L)	Power	7.4 ± 2.5
	SHAM	7.1 ± 2.2
	Total	7.3 ± 2.4
Fasting serum insulin (mU/L)	Power	10.1 ± 5.9
	SHAM	11.1 ± 6.7
	Total	10.6 ± 6.3
* HOMA2-IR	Power	2.7 ± 0.96
	SHAM	3.1 ± 1.2
	Total	2.9 ± 1.1
Physical activity level		
PASE score	Power	113.2 ± 61.9
	SHAM	126.2 ± 63.0
	Total	120.0 ± 62.5
Physical function		
Habitual gait speed (m/s)	Power	1.2 ± 0.2
	SHAM	1.2 ± 0.2
	Total	1.2 ± 0.2
Maximal gait speed (m/s)	Power	1.9 ± 0.3
	SHAM	1.9 ± 0.3
	Total	1.9 ± 0.3

Data presented as mean ± SD, unless otherwise stated. * 16 participants were omitted due to insulin therapy. SD = standard deviation, CI= confidence interval, n = number, HbA1c = glycated hemoglobin, mmol/L = millimoles per Liter, mU/L = milliunits Per Liter, HOMA2-IR = Homeostasis model assessment: insulin resistance. PASE = Physical Activity Scale for the Elderly; higher scores indicate higher levels of physical activity [29].

**Table 3:** Comparison of habitual physical activity (PA) in supervised exercise randomized controlled trials.

Study	Age (years)	Supervised	PA Assessment	Aerobic Exercise Effect on Habitual PA	Resistance Exercise Effect on Habitual PA	Combined Aerobic and Resistance Exercise Effect on Habitual PA
Dunstan	60–80	Y	Subjective	-	No change	-
Castaneda	>55	Y	Subjective	-	Increase	-
Church	30–75	Y	Objective	No change	No change	No change
Balducci	40–75	Y	Subjective	-	-	Increase
GREAT2DO	60–83	Y	Subjective	-	No change	-

## Data Availability

Data are stored securely in a University server and the authors can be contacted regarding access if needed. Other analyses and data entry into this dataset are still in progress so the data are not available in the public domain yet.
